# Oscillating Seebeck coefficients in π-stacked molecular junctions†

**DOI:** 10.1039/c8ra04698k

**Published:** 2018-07-10

**Authors:** Mohsin K. Al-Khaykanee, Ali K. Ismael, Iain Grace, Colin J. Lambert

**Affiliations:** Department of Physics, University of Lancaster Lancaster LA1 4YB UK c.lambert@lancaster.ac.uk mohsin.kad@gmail.com; Department of Physics, College of Science, University of Babylon Babel Iraq; Department of Physics, College of Education for Pure Science, Tikrit University Tikrit Iraq

## Abstract

When a linear aromatic molecule within a nanogap is bound only to a source electrode, and an adjacent molecule is bound only to a drain electrode, the two molecules can interact *via* pi–pi stacking, which allows electrons to flow from the source to the drain, *via* pi–pi bonds. Here we investigate the thermoelectric properties of such junctions, using mono-thiol oligo-phenylene ethynylene (OPE3)-based molecules as a model system. For molecules which are *para*-connected to the electrodes, we show that the Seebeck coefficient is an oscillatory function of the length *L* of the pi–pi overlap region and exhibits large positive and negative values. This bi-thermoelectric behavior is a result of quantum interference within the junction, which behaves like a molecular-scale Mach–Zehnder interferometer. For junctions formed from molecular monolayers sandwiched between planar electrodes, this allows both hole-like and electron-like Seebeck coefficients to be realized, by careful control of electrode separation On the other hand for *meta*-connected molecules, the Seebeck coefficient is insensitive to *L*, which may be helpful in designing resilient junctions with more stable and predictable thermoelectric properties.

## Abstract 2

When two adjacent molecules are slid across each other, quantum interference causes oscillations in their conductance and Seebeck coefficient.

## Graphical abstract

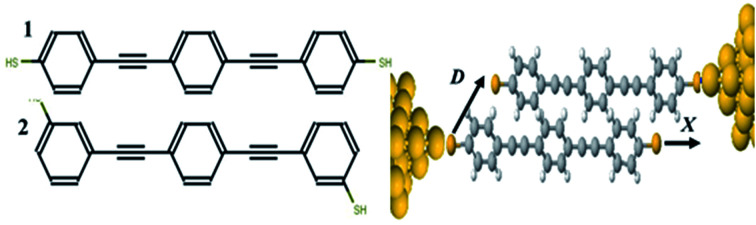

## Introduction

**Fig. 1:**
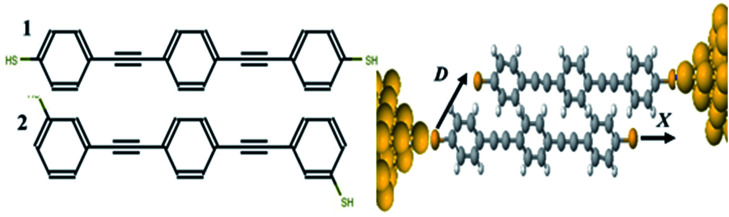
(Left) Molecular structures for oligophenylene ethynylene (OPE3) molecules with thiol anchor groups connected in the *para* (1) and *meta* (2) position. (Right) An example of a stacking geometry for 1 connected between gold electrodes. The parameters *X* and *D* denote the overlap length and separation respectively.

Measurement of the Seebeck coefficient *S* in single-molecule junctions^1–9^ has opened up the possibility of utilizing molecular-scale thin-film materials in flexible thermoelectric devices. A wide variety of single molecules have been measured with reported values of *S* up to 100 μV K^−1^.^9^ A key property determining the sign and magnitude of *S* is the position of the Fermi energy of the electrodes relative to the middle of the HOMO–LUMO gap of the molecule; a positive *S* implies that the Fermi energy *E*_F_ is closes to the highest occupied molecular orbital (HOMO) and a negative *S* means it is closer to the lowest unoccupied molecular orbital (LUMO). Recently it has been demonstrated both experimentally and theoretically that single molecules can display bi-thermoelectric behaviour, in which the sign of *S* changes in response to both geometric changes^10–12^ and the application of pressure,^11^ which shift the positions of the HOMO and LUMO transport resonances relative to *E*_F_. An alternative approach to the control of quantum transport through single molecules involves utilizing quantum interference (QI) of de Broglie waves associated with electrons passing through the molecule,^13–25^ which can increase or decrease the electrical conductance depending on whether QI is constructive or destructive. One type of molecular junction that has shown QI involves two pi-stacked molecules,^26–30^ as shown in Fig. 1, where each molecule is attached to only one electrode by a thiol anchor and electrons flow from one electrode to the other *via* the overlap between the pi-orbitals of the two molecules.

Measurements of the electrical conductance of monothiol-terminated pi-stacked oligo-phenylene ethynylene molecules were first reported in,^26^ while in,^27^ the pi-stacking of these molecules was switched on and off using bulky tertiary butyl groups, which controlled the spacing between the pi systems of adjacent molecules groups. In^28^ it was shown that for pi-stacked junctions formed from two amine-terminated conjugated molecules, the conductance, force and flicker noise differ dramatically when compared with the corresponding monomer junctions, thereby highlighting key differences between intra- and inter-molecular charge transport.

In^29^ conductance oscillations of pi stacked OPE3 with either one (S–OPE3) or two (S–OPE3–S) thiol anchoring groups as a function of their overlap length were measured. Pi-stacking has also been used to design molecular junctions^30^ that either enhance or suppress a phonon transport, while maintaining electrical conductance. In ref. 31 the symmetry breaking effects involved in chemisorbing π-stacked benzene rings to metallic electrodes was shown to have a significant impact on their transport properties. In^32^ five representative pi-stacked systems were studied to show how conjugation length and substituent groups influence their electrical conductance and Seebeck coefficient. These studies clearly demonstrate that the length of the overlap region between the two molecules controls the electronic structure of the junction and the nature of the quantum interference.

In this work we investigate whether the ability to tune the transport properties of such pi-stacked junctions can be used to control their Seebeck coefficient. We show that by varying the length of the pi-overlap region, not only can the magnitude of *S* be increased, but also the molecule can display both positive and negative *S*. This bi-thermoelectric behaviour is attractive, because compatible materials with both positive and negative Seebeck coefficients are needed in the construction of useable thermoelectric modules.^33^

## Theoretical methods

To calculate the electronic transport properties of OPE π-conjugated molecules shown in Fig. 1, we use a combination of the density functional code SIESTA^34^ and the quantum transport code Gollum.^35^ The optimum geometry was calculated for molecules 1 and 2 by relaxing them to a force tolerance of 0.01 eV Å^−1^ using Troullier–Martins pseudopotentials to represent the potentials of the atomic cores,^36^ a generalized gradient approximation (GGA–PBE) functional to describe the exchange correlation,^37^ double-ξ polarized basis set, and a real-space grid was defined with an energy cutoff 150 Rydberg.

In what follows, we study the effect of varying the pi-overlap length, for molecules with either *para* or *meta* connectivities to the electrodes. The optimal stacking geometry for both molecules was calculated initially without the gold electrodes by altering the displacement *X*, and the inter-molecular spacing *D* of junction containing either *para*-connected molecules 1, or *meta*-connected molecules 2, as shown in Fig. 1 (more details are shown in the ESI†) and then relaxing the dimer to minimize the total energy. We define *X* to be the distance between the sulfur atoms along the axis of the molecule, *i.e.* when *X* = 0 the overlap of the molecule is a maximum. In the case of both OPEs 1 and 2 the optimum values found are *X* = 0.161 nm and *D* = 0.33 nm, and this configuration has a binding energy of −0.77 eV (for binding energies a van der Waals^38^ functional was used see Fig. S3 in the ESI†). Within the DFT simulations, each molecule was then attached to one gold electrode as shown in Fig. 1. The electrodes consist of 6 layers of (111) gold each containing 25 gold atoms, and are terminated by a pyramid of gold atoms. The terminal sulfur atom of the thiol group loses it hydrogen atom when it attaches to a gold electrode, resulting in an optimised gold–sulfur binding distance of 2.4 Å. The hydrogen atom of the unattached thiol group remains bound to the sulfur. The zero-bias transmission coefficient *T*(*E*), which is the probability for an electron of energy *E* of electrons to transfer from left-to-right of electrodes^24^ was calculated by using the SIESTA code to obtain the DFT mean-field Hamiltonian, and then using Gollum to compute *T*(*E*). The slope of the natural logarithm of the transmission coefficient then yields the Seebeck coefficient *S via* the relation^39^1
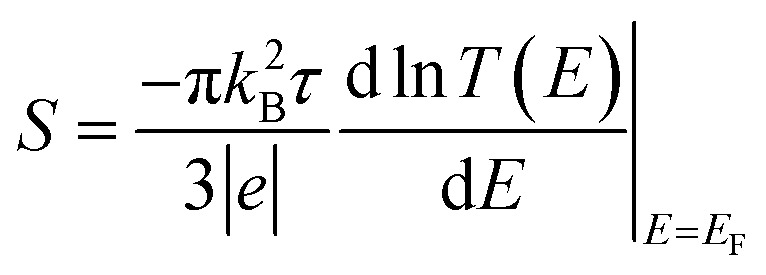
where *k*_B_ is the Boltzmann constant, *τ* is the temperature of the junction, *e* is the electron charge and *E*_F_ is the Fermi level of the gold electrodes.

## Results and discussions

**Fig. 2:**
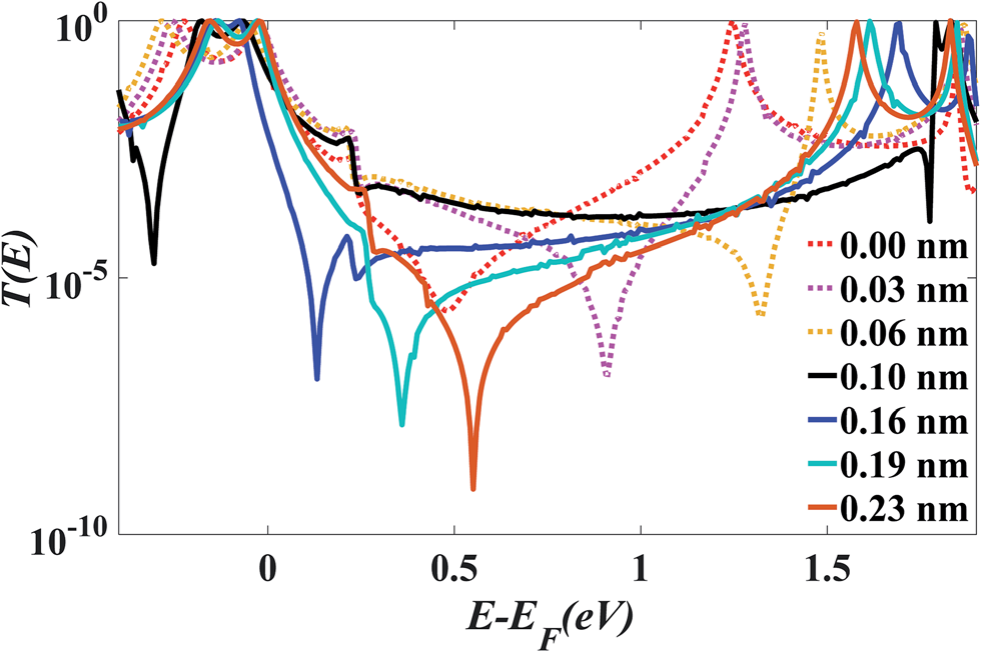
Zero bias transmission coefficient *T*(*E*) of molecule 1 against electron energy *E* for different displacements *X* and fixed separation *D* = 0.33 nm.

After choosing the optimum molecule separation of *D* = 0.33 nm, we varied the displacement *X* of the molecule and calculated the transmission coefficient *T*(*E*). Fig. 2 shows the results for *X* varying between 0 and 0.23 nm for the *para* connected molecule 1, (transmission data for a larger range of *X* are presented in the ESI Fig. S6 and S7†). At a value of *X* = 0.161 nm (which is the optimum stacking geometry) the Fermi energy (*E*−*E*_F_ = 0 eV) lies close to the HOMO resonance, and there is a sharp dip in *T*(*E*) near *E*−*E*_F_ = 0.1 eV in the gap between the HOMO and LUMO transmission resonances. This destructive interference feature is attributed to the multiple transport paths through the stacked molecule, which cause Mach–Zehnder-type interference effects. Fig. 2 shows that as *X* is increased, the anti-resonance moves towards the LUMO resonance and at value of *X* = 0.23 nm the interference dip sits at *E*−*E*_F_ = 0.6 eV. The transmission curves also show that the HOMO–LUMO gap increases as *X* increases, due to the splitting between the two LUMO resonances of the individual molecules, which decreases as the coupling between the two molecules becomes weaker.

**Fig. 3:**
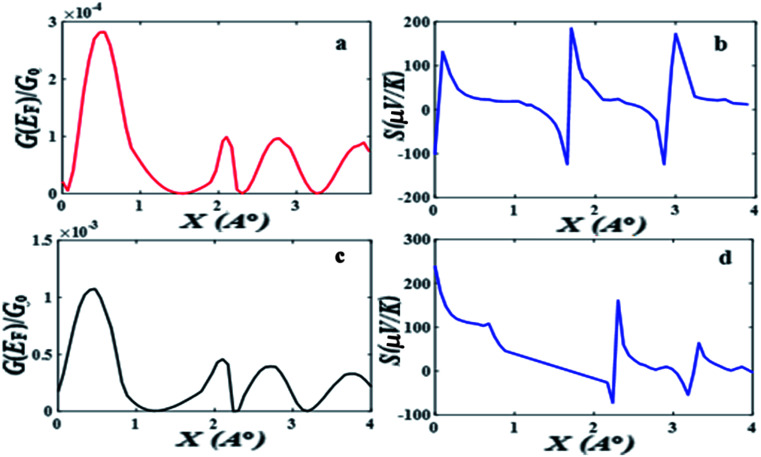
(a) The electrical conductance and (b) the Seebeck coefficient of pi-stacked OPE3s as a function of displacement when *E*_F_ = 0.5 eV relative to the DFT predicted Fermi energy. (c) The electrical conductance and (d) the Seebeck coefficient of pi-stacked OPE3s as a function of displacement when *E*_F_ = 0.3 eV relative to the DFT predicted Fermi energy.

Fig. 2 shows when an anti-resonance passes through the Fermi energy, the gradient of *T*(*E*) changes sign, which demonstrates that the Seebeck coefficient *S* is sensitive to the stacking geometry. Fig. 3 (right panel) shows the calculated value of *S* at room temperature, for a Fermi energy of *E*−*E*_F_ = 0.5 eV (see in Fig. 3(a and b)), *E*−*E*_F_ = 0.3 eV (see in Fig. 3(c and d)), and a separation of *D* = 0.33 nm for values of *X* between 0 and 0.4 nm. At *X* = 0 the sign of *S* is negative and has a magnitude of −100 μV K^−1^. As the displacement *X* is increased the sign of *S* is switched and at a value of *S* at *X* = 0.02 nm is 100 μV K^−1^. The anti-resonance then moves away from the Fermi energy and *S* remains positive with a value of approximately 25 μV K^−1^. At greater displacements the sign of *S* oscillates. Fig. 3 left shows that these oscillations in the sign of *S* are accompanied by oscillations in the electrical conductance.

**Fig. 4:**
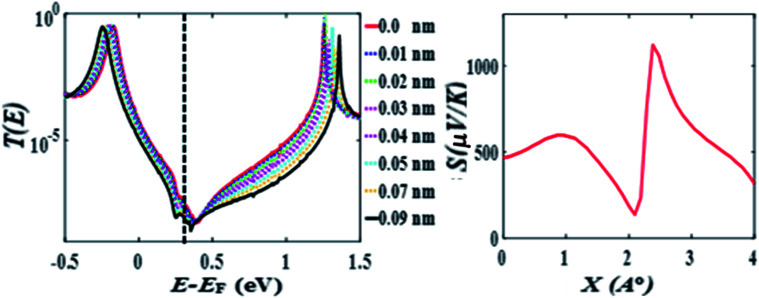
(Left) Zero bias transmission coefficient *T*(*E*) for molecule 2 for different displacements *X* and fixed separation *D* = 0.33 nm. (Right) Seebeck coefficient *S* as a function of overlap length *X* (*D* = 0.33 nm).

The above calculations were repeated for the *meta*-connected molecule 2 and the results are presented Fig. 4 (the separation is *D* = 0.33 and the displacement *X* is varied between 0 and 0.09 nm). Here the transmission *T*(*E*), shows an anti-resonance at 0.4 eV, which does not move across the HOMO–LUMO gap as *X* is increased. The value of the Seebeck coefficient is therefore positive for all values of *X* between 0 and 0.4 nm (see Fig. 4 right panel). This demonstrates that quantum interference effects in the *meta*-connected of molecule 2 are less sensitive to the pi–pi overlap displacement.

## Conclusions

In a previous study of pi-stacked phenyl and anthracene derivatives^32^ it was shown that the Seebeck coefficient can change sign by displacing the molecules both parallel and perpendicular to the direction of current flow. Here, we show similar behaviour in linear pi-stacked OPE3 molecules with *para* connectivity to the electrodes, which have been shown to form pi-stacked junctions experimentally,^26^ but no such oscillatory behaviour occurs for *meta*-connected OPEs. When molecules are *para*-connected to the electrodes, the location of an anti-resonance due to destructive quantum interference is sensitive to the displacement of the molecules and causes the Seebeck coefficient to alternate in sign. This novel bi-thermoelectric behaviour has the potential to inform the design of thin-film thermoelectric materials and devices based on self-assembled molecular layers, where the spacing between planar electrodes could be controlled by introducing electrically insulating molecules of known length into a film of electrically-active pi–pi stacked molecules, thereby tuning the sign of their Seebeck coefficient. This is important technologically, because junctions with Seebeck coefficients of both signs are needed to boost the thermovoltage. On the other hand when molecules are *meta*-connected to the electrodes, the anti-resonance is less sensitive to the displacement of the molecules and the Seebeck coefficient is consistently positive. This is useful in the design of stable junctions with predictable Seebeck coefficients, since small fluctuations in the electrode separation have little effect.

## Conflicts of interest

There are no conflicts of interest to declare.

## Supplementary Material

RA-008-C8RA04698K-s001
