# Supplementary material for: Oscillating Seebeck coefficients in π-stacked molecular junctions
Source: RSC Adv. 2018 Jul 10;8(44):24711–5. doi: 10.1039/c8ra04698k (PMC9082453; doi:10.1039/c8ra04698k)
Supplement: RA-008-C8RA04698K-s001 [file RA-008-C8RA04698K-s001.pdf]

Electronic Supplementary Material (ESI) for RSC Advances.
This journal is © The Royal Society of Chemistry 2018

Supplementary Information

Oscillating Seebeck coefficients in π-stacked molecular junctions

Mohsin K. Al-Khaykanee, [a,b]*Ali K. Ismael,[a,c] Iain Grace,[a] Colin J. Lambert, [a]*

[a]*Department of Physics, University of Lancaster, Lancaster LA1 4YB, U.K.*
[b]*Department of Physics, College of Science, University of Babylon, Babel, Iraq.*
[c]*Department of Physics, College of Education for Pure Science, Tikrit University, Tikrit, Iraq.*

Table of Content

1. Geometry of molecules (S- OPE3-S) and their junctions
2. Binding energy of π-stacked molecules on gold
3. Wave function plots for S-OPE3-S
4. Transmission coefficients of (S- OPE3-S) molecules at various displacements X at Fermi energy (E_F)
5. Transmission coefficients of (N- OPE3-N) at various displacements X at Fermi energy (E_F)

1. Geometry of molecules (S- OPE3-S) and their junctions

The DFT code (SIESTA) was used to obtain fully relaxed geometries of the isolated S-OPE3-S molecules, as shown in Figure S1.

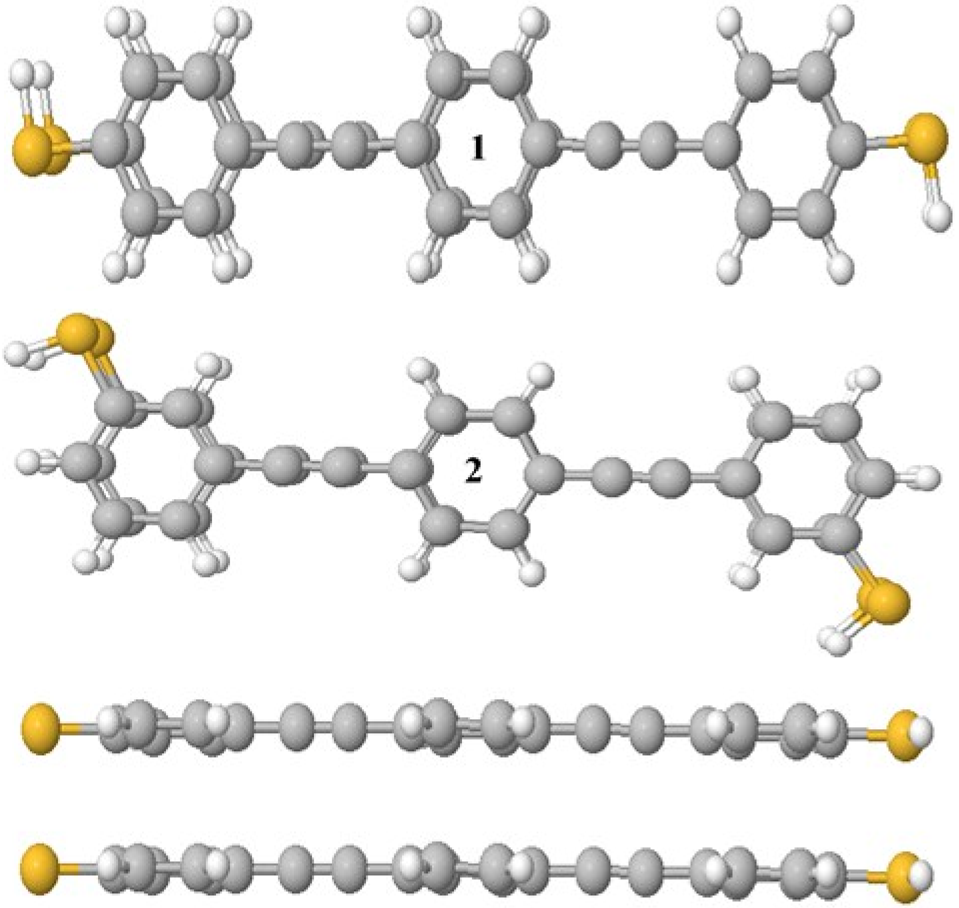

Figure S1 | Fully relaxed isolated S-OPE3-S molecules, para- π-stacked (**1**), meta- π-stacked (**2**), and side view to show π- π stacked molecules

Then S-OPE3-S molecules were connected to gold electrodes as shown in Figure S2 and then further relaxed.

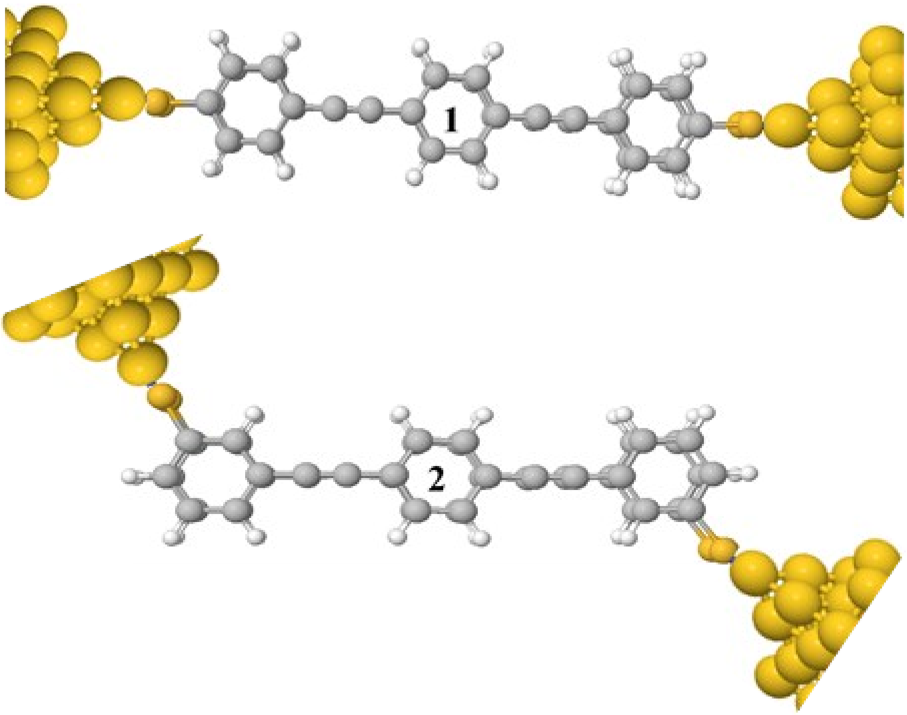

Figure S2 | S-OPE3-S molecule in junctions, para-π-stacked (**1**), meta-π-stacked (**2**)

2. Binding energy of π-stacked molecules on gold

To find the optimum geometry of molecules **1** and **2** we calculated the position of maximum binding energy and to do this we use SIESTA and the counterpoise method, which removes basis set superposition errors (BSSE). One of the molecules is defined as entity A and the other as entity B. The ground state energy of the total system was then calculated using SIESTA and is denoted E_{AB}^{AB}. To do this we use the DFT parameters defined in the main text, but use a Van

der Waals functional[1] instead of LDA to describe the exchange correlation. The energy of each entity was then calculated in a fixed basis, which was achieved through the use of ghost atoms in SIESTA. Hence, the energy of each molecule in the presence of the fixed basis is defined as E_{A}^{AB} and E_{B}^{AB}. The binding energy was then calculated using the following equation:

$$Binding\ Energy = E_{AB}^{AB} - E_{A}^{AB} - E_{B}^{AB} \quad \text{(S1)}$$

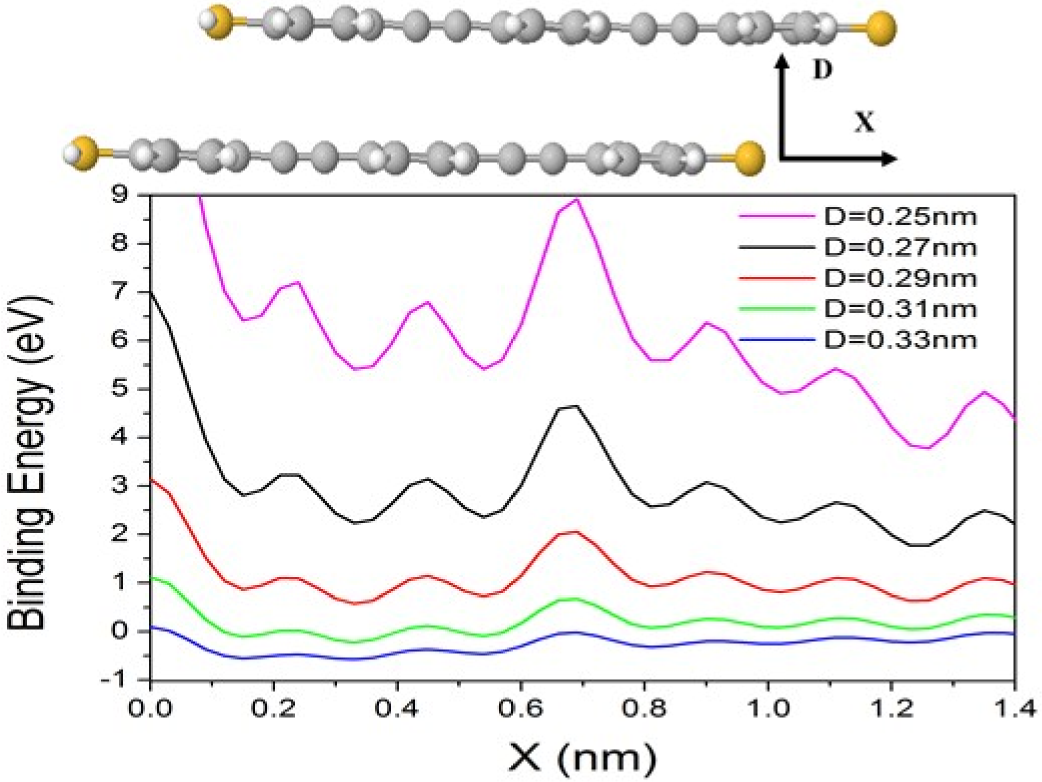

Figure S3 | Binding energy of molecule para-π-stacked (**1**) as a function of parameters *X* and D

3. Wave function plots for S-OPE3-S

The plots below show isosurfaces of the HOMO, LUMO, HOMO-1 and LUMO+1 of isolated molecules.

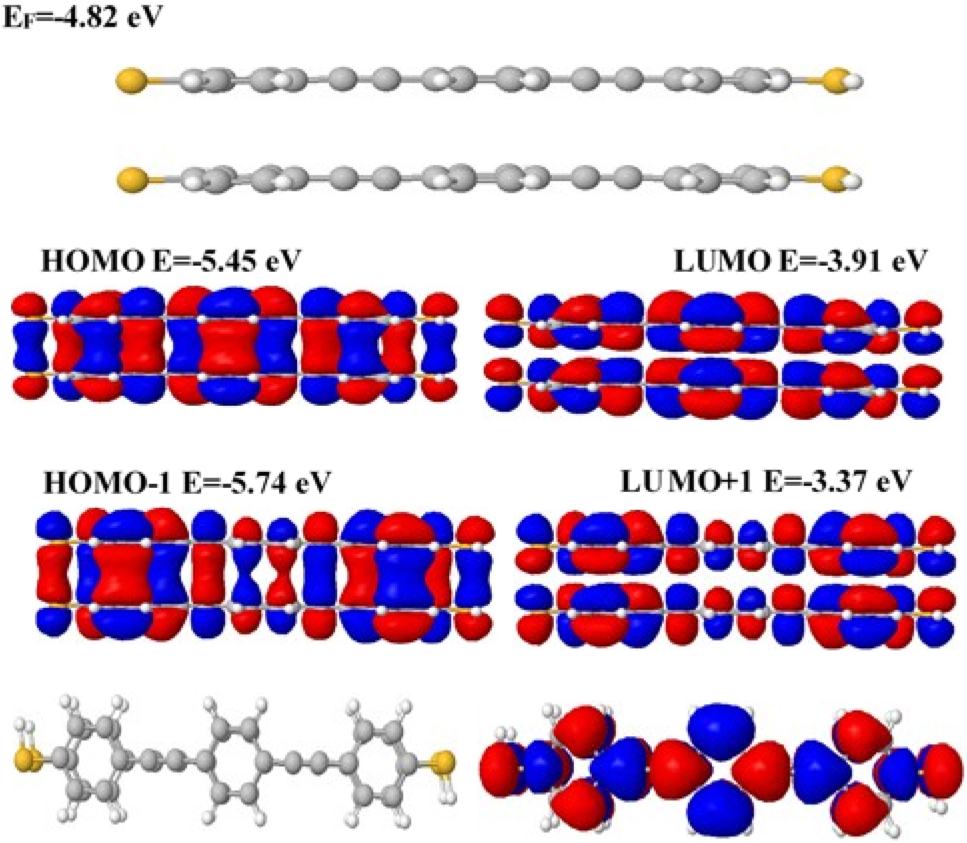

Figure S4 | Wave function for para-π-stacked (**1**) molecule (OPE3). **Top panel**: Fully optimsed geometry of S-OPE3-S. **Lower panel**: HOMO, LUMO, HOMO-1 and LUMO+1 with their energies and top view of **1** molecule

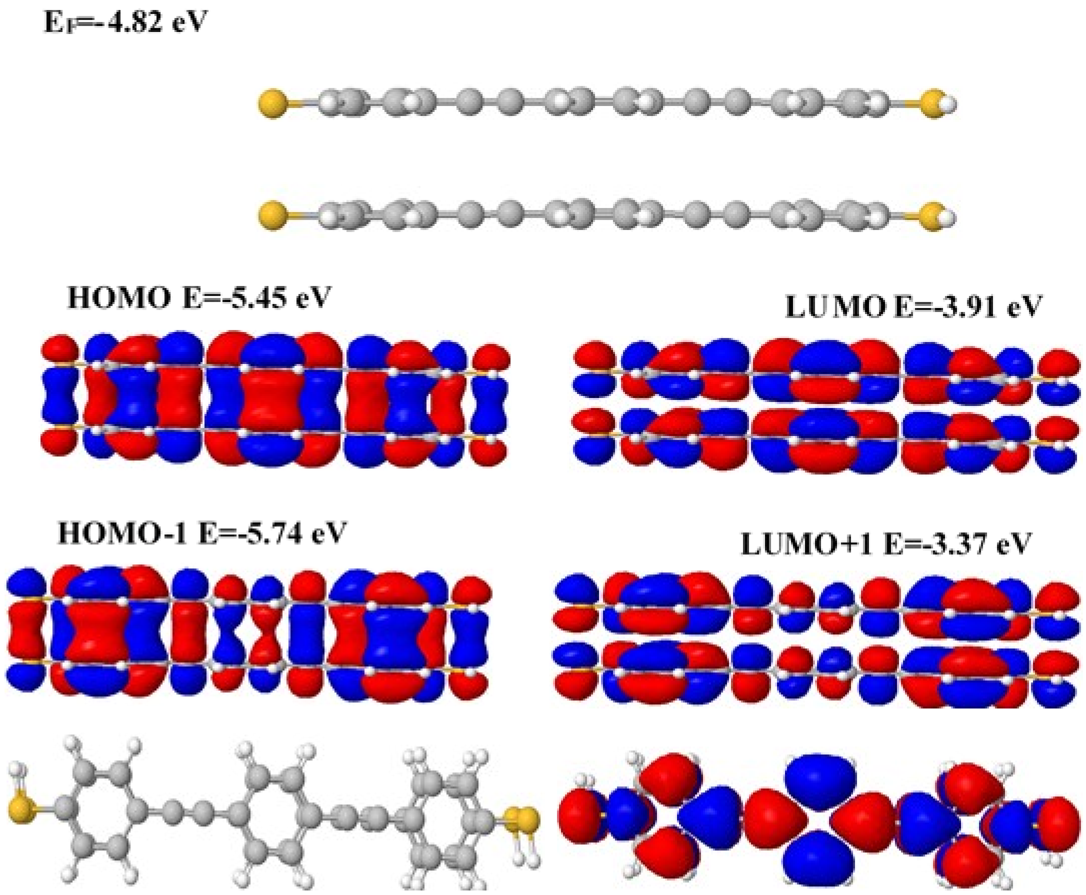

Figure S5 | Wave function for meta-**π-**stacked (**2**) molecule (OPE3). **Top panel**: Fully optimsed geometry of S-OPE3-S. **Lower panel**: HOMO, LUMO, HOMO-1 and LUMO+1 with their energies and top view of **2** molecule

4. Transmission coefficients of (S- OPE3-S) molecules at various displacements *X* at Fermi energy (E_F)

In this section, the transmission coefficient *T(E),* calculated for π-π-stacked molecule (**1**) at various dispacements *X* (see top panel) at Fermi energy (E_F). Figure S6 shows how the destrcutive quantum interefence resonance (dip) moves with increasing the displacement (*X*)

which causes changing the sign of thermopower in some caeses such as **(a)** and **(b)** of Fig S6 .

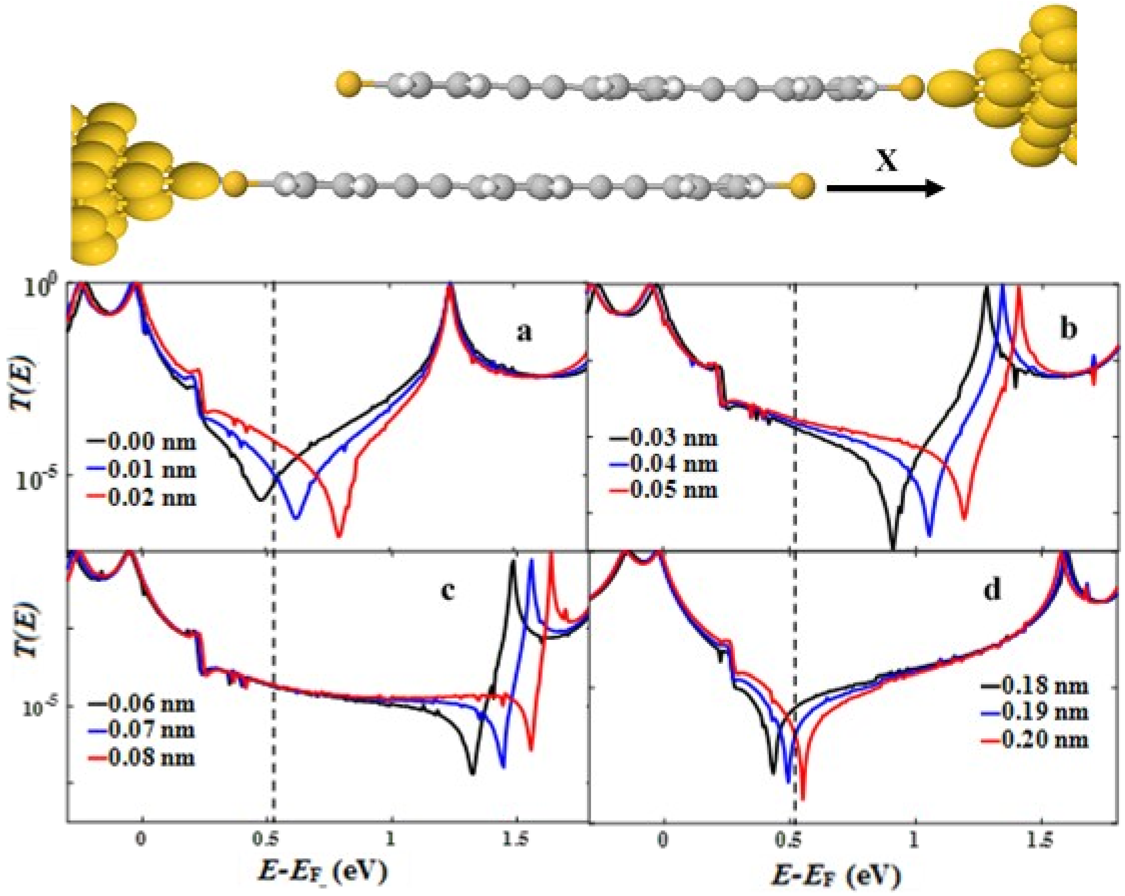

Figure S6 | Illustrates moving of transmission coefficient with displacements *(X*) of π-π stacked S-OPE3-S molecules (**1**) at various distances at Fermi energy (E_F)

To follow the chaning in the sign of thermopower we have varied the displacement from *X* from 0.0 to 0.23 nm. Figure S7 shows how the destrcutive quantum interefence resonance (dip) moves with increasing the displacement *X* .

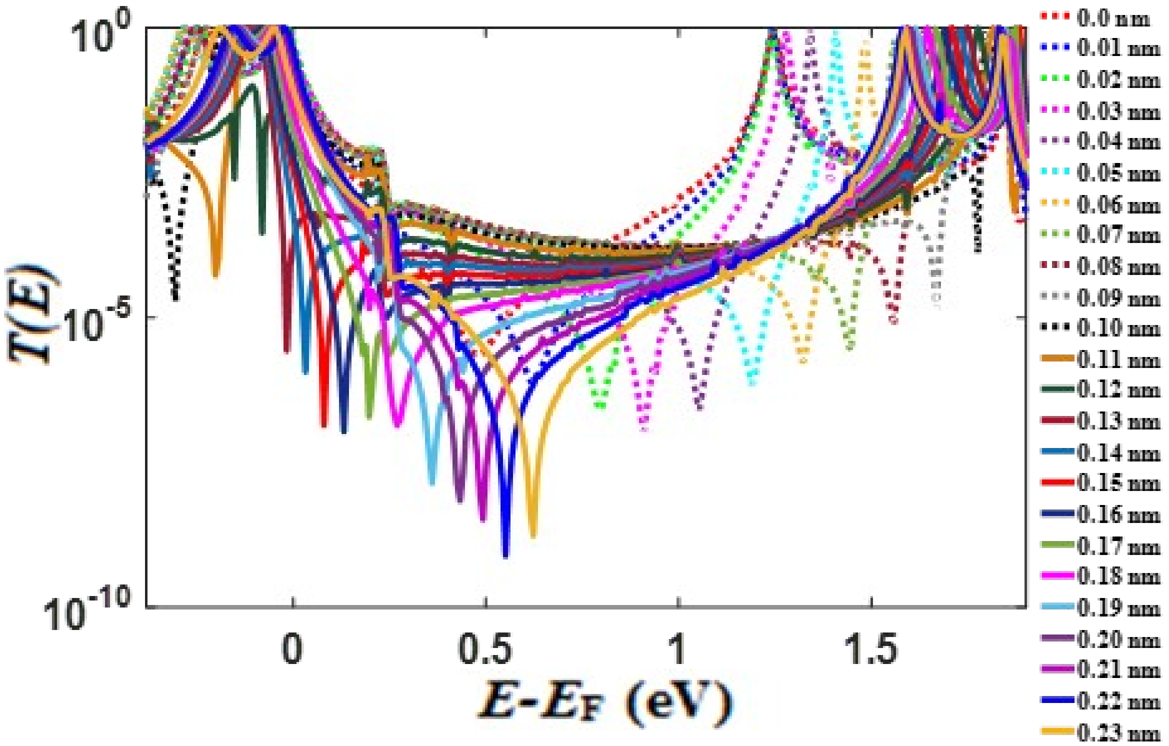

Figure S7. Zero bias transmission coefficient *T(E)* against electron energy *E* for different overlap lengths *X* and fixed separation *D* = 0.33nm (full curves of Fig2 main text).

5. Transmission coefficients of (N- OPE3-N) molecules at various displacements X at Fermi energy (E_F)

Here we repeating the same process in section 4, but with different molecule (anchor groups are nitrogens). The transmission coefficient *T(E),* calculated for pi-pi-stacked molecule at

various dispacements X (see top panel) at Fermi energy (E_F). Figure S7 shows destructive quantum interference resonance (dip) at E-E_F =-1.2 eV same as Fig S6. However the anti-resonance does not move with changing X, which indicates no change in the sign of thermopower. The conductance decreases with increasing the displacement X as shown in Fig S7.

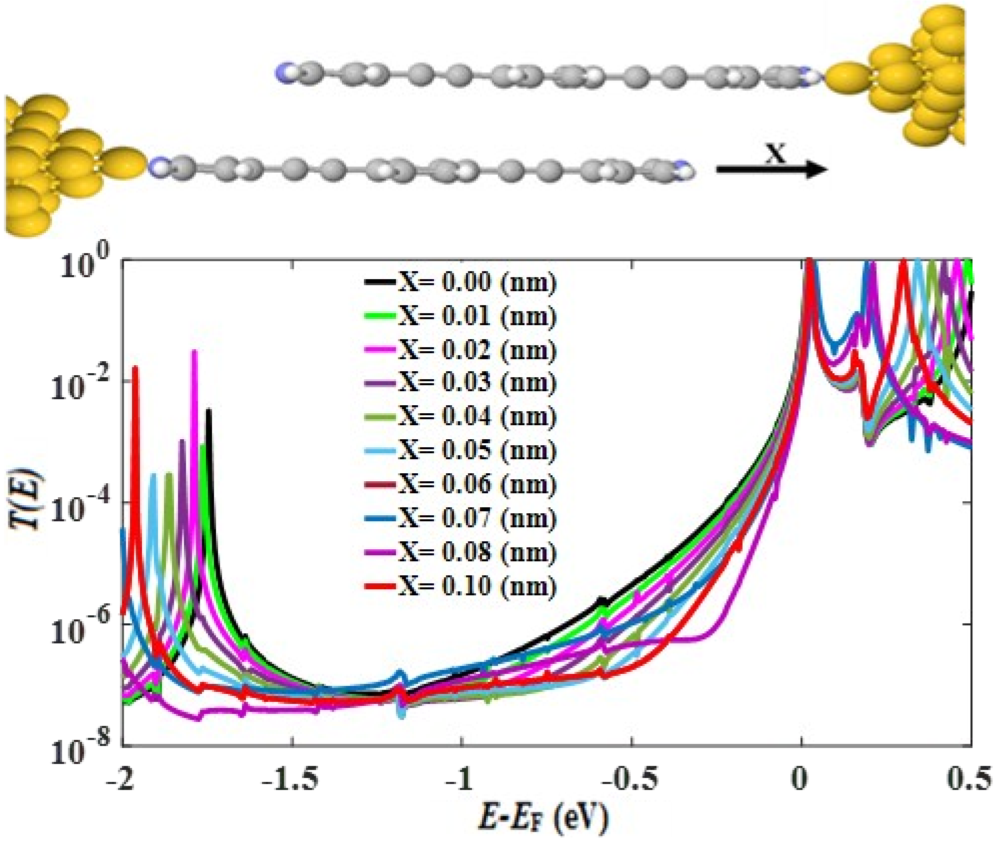

Figure S7 | Illustrates Transport calculations for π-stacking configurations of N-OPE3-N molecule of phenyl ring para (p) connectivity a function of electrons energy. Also, the drops in transmission curves observed for N-OPE3-N result from quantum interference effects and occur when displaced intermolecular π-π stacking molecules.

References:

[1] Dion, L.; Rydberg, H.; Schroder, E.; Langreth, D. C.; and Lundqvist, B. I. Van derWaals Density Functional for General Geometries. Physical Review Letters, 2004, 92(24), pp. 22-25
